# Eastern oysters alter inducible defense mechanism of shell strengthening with age

**DOI:** 10.1242/jeb.250143

**Published:** 2025-07-07

**Authors:** Sarah H. Roney, Gary H. Dickinson, Benjamin A. Belgrad, Marc J. Weissburg

**Affiliations:** ^1^ Georgia Institute of Technology, School of Biological Sciences, Atlanta, GA 30332, USA; ^2^ Brook Byers Institute for Sustainable Systems, Atlanta, GA 30332, USA; ^3^ The College of New Jersey, Department of Biology, Ewing, NJ 08628, USA; ^4^ Dauphin Island Sea Lab, Dauphin Island, AL 36528, USA

**Keywords:** Predator–prey interactions, Phenotypic plasticity, Shell formation, Blue crabs, *Crassostrea virginica*

## Abstract

Eastern oysters, *Crassostrea virginica*, use inducible defenses in the form of strengthened shells to reduce their risk of predation. Inducible defenses often have trade-offs between the costs and benefits associated with the organism's fitness, as developing defenses requires energetic resources. Shell strength is a product of the amount of material laid by the animal (thickness) and the material properties of the shell (e.g. hardness and fracture resistance). Previous studies have suggested that oysters may trade off between shell thickness or shell hardness as a mechanism for increasing shell strength against predation, which are hypothesized to have different energetic requirements. The present study analyzed the shell structural (thickness of composite layers) and micromechanical properties (microhardness and crack propagation tested within individual shell layers) of predator-induced and non-induced juvenile oysters at 4 and 8 weeks post-settlement to determine which shell strengthening mechanism oysters use in response to predator cues. Younger juveniles did not display any significant differences in micromechanical shell properties or shell thickness as a result of induction, though some marginal differences were detected. In contrast, older juveniles use a combination of increased hardness and thickness in shell defense, where induced oysters were 33% thicker overall and 12% harder within their outer prismatic layer. This suggests that oysters alter shell strength using multiple defense mechanisms depending on age, and we postulate that animals switch mechanisms when necessary to balance the advantages and associated costs of defense with an individual's physiological needs for growth.

## Teaser

**Summary:** Eastern oysters strengthen their shells to reduce predation risk, combining increases to shell hardness and thickness as a strengthening mechanism.

## INTRODUCTION

In many predator–prey systems, prey use inducible defenses to reduce the risk of predation and increase their likelihood of survival to reproductive age. These defenses can be biochemical (Baldwin, 1998), morphological (Harvell, 1992), behavioral (Smee and Weissburg, 2006) or some combination of these strategies. Inducible defenses often evolve when the timing of predation events is highly variable or defenses have a significant cost (Harvell, 1990). Such costs can occur via energy reallocation to defense as opposed to growth or other activities, or through lost opportunities, where the organism forgoes other benefits owing to their defense (Tollrian and Harvell, 1999). For example, clams cease filter feeding behavior in response to predator stimulus in order to decrease their risk of discovery (Smee and Weissburg, 2006), but may experience a cost to this defense via reduction in their food intake, particularly when resource levels are low (Grizzle and Morin, 1989). The costs of inducible defenses, and their associated trade-offs, can be difficult to determine but can have important consequences on community interactions (Peacor and Werner, 1997). Furthermore, these costs may be balanced or reallocated as environmental or community pressures change (Brönmark and Miner, 1992), but it remains unclear whether individuals may balance the associated costs through different mechanisms (i.e. a trade-off) while maintaining their defensive changes.

The eastern oyster, *Crassostrea virginica*, exhibits one such inducible defense, but the costs or trade-offs of this defense remain unknown. Eastern oysters are important ecosystem engineers with high economic and ecological value (Grabowski and Peterson, 2007; Grabowski et al., 2012). This species is a major prey resource for estuarine ecosystems and is consumed by various crab (O'Connor et al., 2008), fish (Anderson and Connell, 1999) and gastropod predators (Muthiah et al., 1987), which often influences oyster reef distribution and success (Tedford and Castorani, 2022). Oysters have evolved an inducible defense in response to these many predators, where juveniles, also known as spat, strengthen their shells against breaking when exposed to chemical cues in the waste products of their predators over the course of several weeks (Robinson et al., 2014; Eason et al., 2021; Roney et al., 2023). This inducible defense is incredibly effective at reducing predation; juvenile oysters induced with predator cues for 1–2 months exhibit approximately 50% stronger shells (Belgrad et al., 2021) and experience up to 300–600% greater survivorship against natural predators compared with non-induced oysters (Belgrad et al., 2021, 2023). However, the underlying mechanism driving this shell strengthening, and any associated trade-offs in energetic expenditure, is currently unknown.

Shell strength, i.e. the force required to fracture through a shell at the macro-scale, can be altered by several possible mechanisms, including shell size or shape, thickness, microstructural and micromechanical properties of the material, and composition including organic content (Kim et al., 2016). Alterations to shell thickness – or more specifically, animals depositing thicker shell material – have been identified in many shelled organisms, and this method is known to be effective for reducing shell breakage (Zuschin and Stanton, 2001; Avery and Etter, 2006). However, increases to shell hardness (i.e. resistance to plastic deformation) are also known to be important for resisting cracks or fractures in shell material and are often associated with increases in organic content (Carriker, 1996; Zuschin and Stanton, 2001; Newell et al., 2007; Esteban-Delgado et al., 2008). An initial study investigating the mechanism of oysters' morphological defense suggested that strengthened oysters may experience a trade-off between shell calcification and amino acid content owing to limits in the energetic resources available to an individual, implying that juvenile oysters must allocate energetic resources into either a thicker or a harder shell to improve overall strength (Scherer et al., 2018). This inducible defense has only been recorded in juvenile oysters within their first few months of life, and experiments show that the effects of their shell strengthening defense diminish after approximately 7–9 months (Belgrad et al., 2025). Elucidating the mechanism underlying shell strengthening in juvenile oysters may allow us to understand how individuals manage the energetic costs and trade-offs of inducible defenses and how oysters employ these mechanisms as they develop.

Eastern oyster shells are composed of calcium carbonate (CaCO_3_) in the form of calcite and consist of two mineralized layers. The inner layer, known as the foliated layer, is composed of calcitic laths of largely inorganic CaCO_3_ separated by a thin organic matrix (Galtsoff, 1964; MacDonald et al., 2010; Johnstone et al., 2015), and this foliated layer can be thickened in defense by increasing the rate of calcification to deposit more shell material. The outer shell layer, known as the prismatic layer, is composed of calcitic prisms enveloped by organic material (proteins, carbohydrate and lipids) with the thickness of organic matrices much greater than in the foliated layer (Checa et al., 2005; Esteban-Delgado et al., 2008; MacDonald et al., 2010). Organic content in the prismatic layer increases the flexibility of the shell material (Esteban-Delgado et al., 2008), and this improved pliability is associated with increases to the compressive strength (Zuschin and Stanton, 2001; Newell et al., 2007) or microhardness of the material (Carriker, 1996; Prezant et al., 2022). Therefore, it is possible that shelled organisms may alter micron-scale shell hardness by developing shell with more, or different, organic material as a way to improve macro-scale fracture resistance. Precipitation of calcium carbonate is hypothesized to be less energetically costly than the production of organic content (Stechele and Lavaud, 2024); studies estimate that calcification costs roughly 1–2 J mg^−1^ CaCO_3_ (Palmer, 1983), whereas organic material is much more expensive at approximately 29 J mg^−1^ protein synthesis (Palmer, 1992), relative to the total energetic availability within an individual. Further research on the Pacific oyster, *Crassostrea gigas*, indicates that production of proteinaceous material, such as within the prismatic shell, accounts for more than half of the total metabolic energy in larval development (Lee et al., 2016). Therefore, it is possible that shell thickening (i.e. precipitation of more calcium carbonate material) as a defense mechanism may be associated with lower energetic costs to the defended individual relative to the benefits of defense, and may therefore be the preferred defense mechanism compared with increasing shell hardness via changes to organic material.

Understanding the mechanism oysters use to strengthen their shells against predation and the variability of defensive responses as oysters age during predator exposure can provide insight into how organisms minimize developmental costs and manage energetic trade-offs in inducible defenses. We leveraged a previous predator-induction experiment (Belgrad et al., 2021) using shells of preserved juvenile oysters to analyze the structural and micromechanical shell properties of oyster spat from two age groups: 4 weeks and 8 weeks post-settlement. In the study from Belgrad et al. (2021), oysters exhibited 40% and 60% stronger shells (standardized crushing force of respective age groups at the macro-scale) when induced and experienced significantly greater field survival. Our analysis of these shells allowed us to examine the mechanism that juvenile oysters use to strengthen their shell and whether this defense mechanism differs by post-settlement age (or length of cue exposure). We hypothesized that younger oysters may be under greater energetic constraints and therefore use less costly mechanisms (shell thickening) to defend against predation compared with older oysters.

## MATERIALS AND METHODS

### Oyster culturing

The oyster spat used in this experiment were preserved from a previous study on shell strength of predator-induced spat, which confirmed that exposure to predator cues resulted in stronger shells, measured as the amount of force (N) required to break shells standardized to size (Belgrad et al., 2021). Eastern oysters, *Crassostrea virginica* (Gmelin 1791), were spawned and cultured at Auburn University Shellfish Laboratory on Dauphin Island, AL, USA, in May 2019 using ∼80 individual broodstock. Oyster larvae were settled onto sun-bleached oyster shell, where they metamorphosized into spat-on-shell, and were housed in four flow-through holding tanks (2.4×0.9 m) filled to a water depth of 0.4 m and flowing at a rate of 36.9 l min^−1^ with natural seawater from Mobile Bay, AL, USA. Oyster spat-on-shell were randomly placed in seven oyster aquaculture baskets (∼140 adult shells per basket, 20,000 spat per tank) spaced evenly along the length of the tank and suspended within the tanks to avoid sediment smothering the spat (28 baskets, 80,000 spat total). Two of the holding tanks were kept with only oysters to serve as controls (non-induced), whereas the two treatment tanks also each held four caged live adult blue crabs (*Callinectes sapidus*, Rathbun) to continuously provide predator cues to the tanks (induced). These crabs were fed one adult oyster (∼5.0 cm in length) daily and were replaced with healthy, fresh caught crabs at least biweekly. Oyster cages were rotated daily around crab cages to reduce differences in growth owing to proximity to cue sources or water intake. A subset of four natural spat-on-shell clusters were removed from each tank after 4 and 8 weeks of culturing under these conditions: two random clusters from the cage in the tank center and two random clusters from the cage next to the tank edge nearest the drain, representing the maximum distance between any cages in the tank. Spat-on-shell clusters were stored in 70% ethanol, following the methods of Beniash et al. (2010) and Dickinson et al. (2012). Preservation of oysters was necessary for our experimental approach, which employed a large number of replicate juveniles; supporting work showed that ethanol preservation does not affect the micromechanical properties tested here (Supplementary Materials and Methods, Fig. S1, Table S1).

### Sample preparation

Oyster spat-on-shell were removed from ethanol solution in March 2023. A total of five left (top) shell valves from each nursery tank per induction state and age group (*n*=10 per induction state and age group; 40 valves total) were carefully removed from individual spat and any soft tissue was removed using forceps and scalpel. Individual valves were haphazardly selected from clusters to ensure at least one spat was sampled from each cluster. The separated shell valves sat in 100% ethanol overnight to assist with removal of tissue remains, then were rinsed with DI water, dried at room temperature for 2–3 h and finished drying in a low-temperature vacuum oven at 45°C and 25 Hg mm for 2 h. The shell valves were then mounted and polished following standard techniques (Prezant et al., 2022). Each valve was placed in a 32 mm mounting cup, with the ventral edge of the shell affixed to the base of the cup using a coil mounting clip that had been glued to the bottom of the cup. This was left to dry overnight, before mounting with Bisphenol A Epichlorohydrin epoxy and hardener mixed in a 10:3 ratio (Allied High Tech). After the epoxy hardened for 24 h, the mounted valves were removed from their mounting cups and ground to a plane that was visually approximated near the center of the shell running along the longest axis from anterior to posterior end. The shell valves were then polished to 0.04 µm using polycrystalline diamond solution and colloidal silica suspension (Allied High Tech).

Panoramic images of each shell sample were taken under polarized light using a reflected light microscope (Zeiss Axioscope.A1 with a Zeiss, AxioCam 105 color camera), with the analyzer set to 10 deg. Panoramas were constructed using imaging software (Zeiss Zen 3.8). Four-week-old spat valves were imaged under a 5× objective, and 8-week-old spat valves were imaged under a 2.5× objective.

### Determining shell thickness

Measurements of the thickness of the foliated layer, the prismatic layer and total shell thickness took place using ImageJ FIJI 1.54f (Schindelin et al., 2012) for both 4- and 8-week-old oysters of both induction states. Panoramic images had a grid placed over them (100 µm^2^ for 4-week-olds, and 200 µm^2^ for 8-week olds) and images were divided approximately into thirds. All thickness measurements were taken from the middle third of the shell valve image, with one measurement per grid cell for the foliated layer, prismatic layer and total thickness, resulting in measurements approximately every 100 µm (*n*=19–31 per 4-week-old shell, and *n*=16–22 measurements per 8-week-old shell, depending on overall spat size).

### Determining shell microhardness

Microhardness testing was performed with a Mitutoyo HM-200 microhardness testing machine. The total size (hinge to umbo) of each shell valve was measured using digital calipers (Mitutoyo Absolute Digimatic) to the nearest 0.01 mm. Microhardness was measured across approximately 10 indents per shell layer conducted on each shell sample (Table S2), spaced approximately evenly across the middle third region of the shell (200–700 µm apart, depending on the size of each shell). All indents were performed with a 5 g load with a 5 s approach time, 5 s dwelling time and 5 s unload time. An image of each indent was taken using a Zeiss Axiocam 305 color microscope, and the diagonals of each indent (µm) were measured using Zeiss Zen 3.6 Blue edition software (Fig. S2). The Vickers microhardness number (VHN) was determined as 1.8543*F*/*d*^2^, where *F* is force (kg) and *d* is diagonal length (mm), as described by ASTM International (ASTM, 2019). During image analysis, the number of cracks and the length of the longest crack emanating from each Vickers microhardness indentation was recorded. Crack length (µm) was measured by placing a circle at the centermost point of the Vickers microhardness indentation and measuring circle radius to the end of the longest crack whose source could be traced back to the indentation (Fig. S2; Anstis et al., 1981; Baldassarri et al., 2008).

### Statistical analysis

As outlined in the ASTM standard (ASTM, 2019), microhardness tests were removed from the analysis if the indent was kite-shaped, with one diagonal more than 10% larger than the other, or were indented into an unreliable position on the shell, such as between prisms or within an unacceptable range (less than 1× the average diagonal length) from the edge (*n*=7–10 per shell; Table S2).

All statistical analyses were performed in RStudio 4.3.1. The effects of induction status and age were assessed with generalized linear mixed effects models (GLMM) with the glmmTMB package (Brooks et al., 2017) using a gamma distribution with a log link. Separate models were run for each dependent variable measured: foliated layer thickness, prismatic layer thickness, overall shell thickness measurements, foliated microhardness, prismatic microhardness, foliated crack size and prismatic crack size. Models included the interaction between induction state and age as a fixed effect. Individual shell ID nested within nursery tank were included as random effects to account for potential variation in culture conditions, and repeated measures across shell samples. Overall shell length was assessed using the same methodology, except with only nursery tank included as a random factor, as only one total shell size measurement was collected per shell. The data on number of cracks for both the foliated and prismatic layers were also analyzed similarly, with the only difference being that GLMMs used a Poisson distribution. A Wald Type II chi-squared test was performed on each model to determine significant differences in data based on the explanatory factors. If differences were detected in more than one factor, within group pairwise comparisons were conducted to distinguish differences between the levels of both factors using a test of estimated marginal means from the emmeans package (https://CRAN.R-project.org/package=emmeans). Data are presented using boxplots where appropriate, but mean±s.e.m. values are shown in Table S3.

## RESULTS

### Oyster size increases with age

**Fig. 1.**
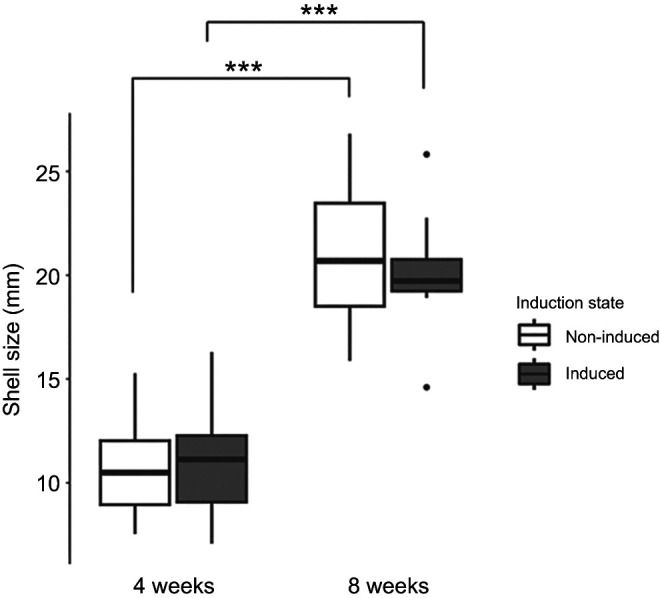
**Shell size (mm) of predator induced and non-induced oysters of 4 and 8** **weeks of age measured from the present study (*n*=10 per age and induction state).** The boxplot displays median and the first and third quartiles of the data and black dots display data points that fall outside of this range. Age strongly affected size (Type II Wald chi-square test on GLMM with age and induction state as fixed factors), where 8-week-old oysters were significantly larger than 4-week-old oysters. Within-group *post hoc* comparisons were made using estimated marginal means and brackets indicate significant differences between groups (***P<0.001).

The size of oysters was affected by age (Type II Wald chi-square test, χ^2^=110.9, *P*<0.001; Fig. 1,), but not induction or the combination of the two factors. Older oysters were significantly larger than younger oysters, displaying an approximately 62% larger size. There were no effects of induction on the size of the oysters (*n*=40) in either age group included in this study.

### Older juvenile oysters adjust shell thickness to defend against predation

**Fig. 2.**
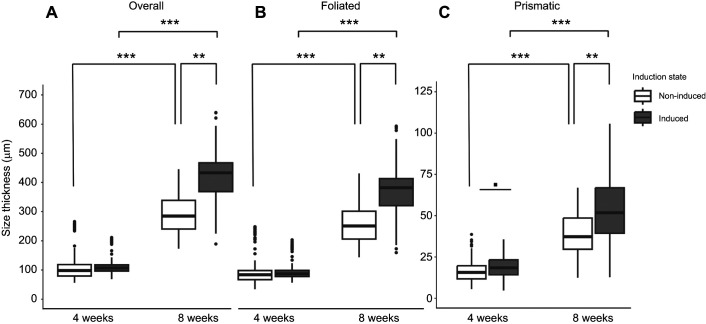
**Thickness of shell layers in induced and non-induced oyster spat-on-shell of two ages.** (A) Overall, (B) foliated and (C) prismatic. The boxplot displays median and the first and third quartiles of the data, and black dots display data points that fall outside of this range. Data were collected from 19–31 measurements (depending on size) on 10 replicate shells per age and induction state. Older, 8-week-old oysters grew significantly thicker shells when induced with predator cues compared with non-induced oysters of the same age. Four-week-old oysters did not exhibit any significant differences between induced and non-induced oyster thickness. Statistical analysis (Type II Wald chi-square test on GLMM with age and induction state as fixed factors) revealed shell thickness, overall and each individual layer, was affected by age, induction and their interaction. Within-group *post hoc* comparisons were made using estimated marginal means and asterisks indicate significant differences between groups (**P<0.01, ***P<0.001).

Overall shell thickness was affected by oyster age (Type II Wald chi-square, χ^2^=205.9, *P*<0.0001; Fig. 2A) and induction state (χ^2^=4.97, *P*<0.001), but not the interaction between age and induction (χ^2^=2.63, *P*=0.1046). Overall, 8-week-old oysters were 102% thicker than 4-week-old oysters when comparing the two age groups (emmeans, *z*-ratio=−8.9, *P*<0.001 for non-induced, *z*-ratio=−11.3, *P*<0.001 for induced; Fig. 2A). There also were strong thickness differences owing to induction within older oysters, but these induction-related thickness differences were not present in younger oysters. Induced 8-week-old oysters were 34% thicker than their non-induced counterparts (emmeans, *z*-ratio=−2.8, *P*<0.01), whereas 4-week-old induced oysters were not different compared with non-induced oysters (Fig. 2A). This indicates that although shell thickening is a natural process of oyster aging, induction further increases shell thickness as oysters age.

These trends were also observed when foliated and prismatic shell layers were assessed separately, where both individual layers were thicker for older induced oysters and the effects of induction were strong for older oysters (Fig. 2B,C). The foliated layer thickness was a function of age (Type II Wald chi-square test, χ^2^=197.5, *P*<0.0001; Fig. 2B) and induction status (χ^2^=3.92, *P*<0.05), whereas the interaction between age and induction state was not significant (χ^2^=2.84, *P*=0.09). Similarly, prismatic layer thickness was also a function of age (Type II Wald chi-square test on GLMM, χ^2^=257.2, *P*<0.0001; Fig. 1C) and induction state (χ^2^=16.1, *P*<0.001), but not the interaction between both factors. Within the 8-week-old age group, induced oysters' foliated and prismatic shell layers were both 33% thicker than those of non-induced oysters (emmeans, *z*-ratio=−2.6, *P*<0.01 for foliated, *z*-ratio=−3.9 *P*<0.001 for prismatic; Fig. 2B,C). In the 4-week-old oysters, there were no significant differences in thickness of the foliated layer due to induction (Fig. 2B), though the prismatic layer of induced oysters was 7% thicker than that of non-induced oysters, though this was not significantly different (emmeans, *z*-ratio=−1.8, *P*=0.0683; Fig. 2C). The ∼33% thickness increases overall and within both shell layers of 8-week-old induced oysters again implies that induction results in significant shell thickening as a result of exposure to predator cues (Fig. 2).

### Microhardness alterations depend on shell layer

**Fig. 3.**
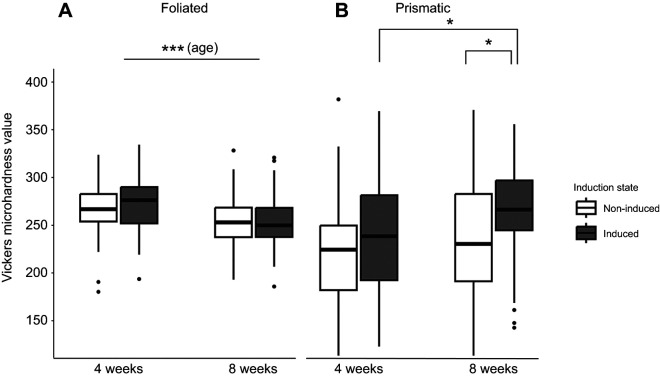
**Vickers microhardness value of shell layers of 4- and 8-week-old oyster spat-on-shell.** (A) Foliated and (B) prismatic. The boxplot displays median and the first and third quartiles of the data and black dots display data points that fall outside of this range. Data were collected from 7–10 measurements (Table S2) on 10 shells per age and induction state. Within the foliated layer, oyster age, but not induction status, affected shell microhardness (A, Type II Wald chi-square test on GLMM with age and induction state as fixed factors), where 4-week-old oysters were harder than 8-week-old oysters. The prismatic layer was affected by both age and induction, where older, induced oysters were significantly harder compared with non-induced oysters (B, Type II Wald chi-square test on GLMM with age and induction state as fixed factors). Brackets represent significant difference based on overall effect of age (A) or within-group *post hoc* comparisons made using estimated marginal means (B). Asterisks indicate significant differences between groups (*P<0.05, ***P<0.001).

Shell microhardness within the inner, foliated layer of oyster spat was influenced by age (Type II Wald chi-square test on GLMM, χ^2^=14.7, *P*<0.001; Fig. 3A), but not induction state or the interaction between them. Comparison of the foliated layer hardness between 4- and 8-week-old-oysters revealed that younger oysters overall were 6% harder than older oysters. Specifically, 4-week-old induced oysters were 7% harder than older oysters, and younger non-induced oysters were 5% harder than their older counterparts (Fig. 3A), indicating that foliated layer hardness decreases as oyster spat age regardless of predator cue induction.

Conversely, shell microhardness within the outer prismatic layer of oysters was affected by induction state (Type II Wald chi-square test on GLMM, χ^2^=7.0, *P*<0.01; Fig. 3B) and age (χ^2^=5.4, *P*<0.05), with no significant interaction between the two factors. When comparing the prismatic layer hardness within age groups, younger oysters were 8% harder than non-induced oysters of the same age, a trend that was not statistically significant, whereas older induced oysters were 12% harder than their counterparts of the same age (emmeans, *z*-ratio=−2.3, *P*<0.05 for the 8-week-old group; Fig. 3B). This indicates that predator chemical cues are inducing older oyster spat to harden the prismatic layer of their shells. Within the induced group, 8-week-old oysters were significantly harder by 10% than 4-week-old oysters (emmeans, *z*-ratio=−2.201, *P*<0.05; Fig. 3B), which also indicates that older oysters produce much harder shell material as a result of induction.

### Induction and age improve the structural integrity of prismatic shell material

**Fig. 4.**
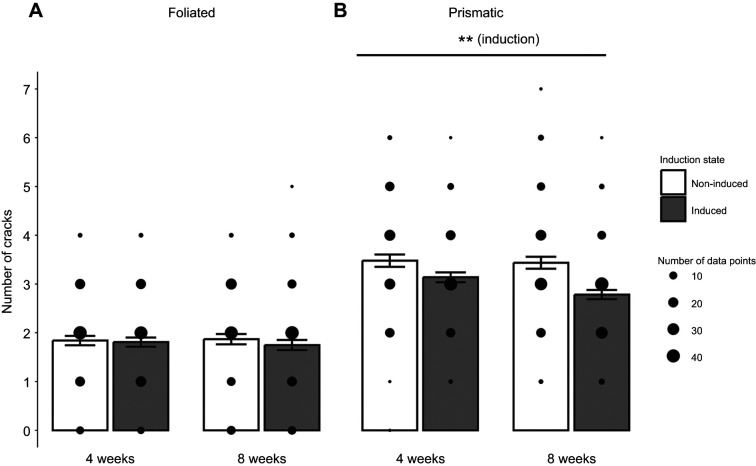
**The mean±s.e.m. number of cracks appearing after Vickers microhardness indent test on shell layers.** (A) In the foliated layer, there were no differences in the number of cracks. (B) In the prismatic layer, induction state significantly affected the number of cracks (Type II Wald chi-square test on GLMM with age and induction status as fixed effects), where older induced oysters experienced significantly fewer cracks overall. Brackets represent significant difference based on overall effect of induction (B) and asterisks indicate significant differences between groups (**P<0.01). Data were collected from 7–10 measurements (Table S2) on 10 shells per age and induction state. Individual data points are displayed, where the size of the point indicates the number of individual data points represented.

**Fig. 5.**
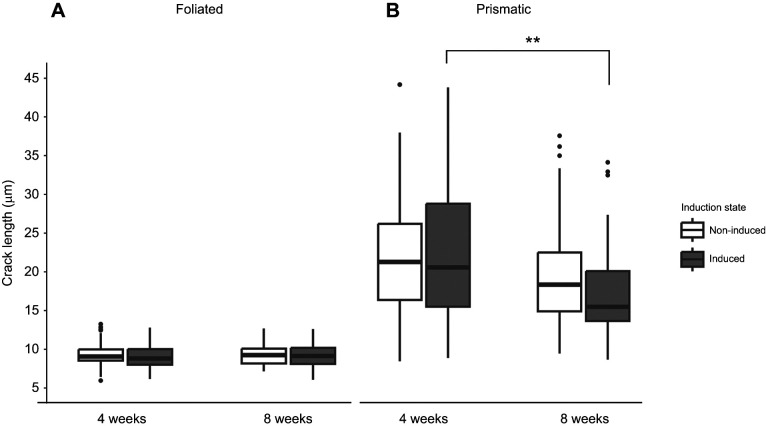
**The length of cracks appearing after Vickers microhardness indent test on shell layers.**  (A) Folidated and (B) prismatic. The boxplot displays median and the first and third quartiles of the data and black dots display data points that fall outside of this range. Data were collected from 7–10 measurements (Table S2) across 10 shells per age and induction state. The length of cracks in the foliated layer (A) was unaffected by age or induction, but crack size in the prismatic layer (B) was significantly smaller for older oysters (Type II Wald chi-square test on GLMM with age and induction status as fixed effects). Asterisks indicate significant differences between groups (**P<0.01).

The cracking frequency within the foliated layer was not affected by induction state, age or any interaction of the two factors (Type II Wald chi-square test on GLMM; Fig. 4A). The length of cracks in the foliated layer was also unaffected by induction state, age or any interaction (Type II Wald chi-square test on GLMM; Fig. 5A).

Conversely, the prismatic layer of induced oysters experienced fewer and smaller cracks compared with non-induced oysters. The number of cracks that appeared in the prismatic layer of oysters was significantly affected by induction state (Type II Wald chi-square test on GLMM, χ^2^=7.12, *P*<0.01; Fig. 4B), but not age or any interaction between the two. Overall, induced oysters experienced 15% fewer cracks compared with non-induced oysters. Specifically, the 8-week-old age group exhibited 20% fewer cracks and the 4-week-old group exhibited 10% fewer cracks (Fig. 3B). Crack length in the prismatic layer of oysters was influenced by age (Type II Wald chi-square test, χ^2^=11.0, *P*<0.01; Fig. 5B), but not induction state or any interaction effect of these factors, and older oysters exhibited approximately 18% smaller cracks overall compared with younger oysters.

## DISCUSSION

Our examination of the shell strengthening defense mechanisms in eastern oysters revealed that alterations to shell hardness and thickness in response to predator cues are highly dependent on animal age and vary depending on the shell layer. Oyster shell thickness was significantly greater for older oysters but was also heavily influenced by exposure to predator cues; older induced oysters were significantly thicker than non-induced oysters of the same age. Although changes in shell thickness were consistent across shell layers, changes in shell hardness with age and predator cues were more complex: the inner, foliated layer hardness was affected by age, whereas the outer, prismatic layer was influenced by both age and predator exposure. Younger oysters were significantly harder than older oysters within their foliated layer regardless of induction status, whereas older induced oysters exhibited significantly harder prismatic layers as compared with non-induced oysters. Furthermore, the alteration of shell properties in response to predator cues appears to improve the material's resistance to breaking as older induced oysters experienced fewer cracks within their hardened prismatic layer. Age also appears to mitigate cracking size in the prismatic layer, where older induced oysters experienced significantly smaller cracks compared with younger oysters. This cracking pattern was not found in the foliated layer, which similarly did not exhibit any changes in shell hardness in response to predator cues. These findings indicate oysters may not experience a simple trade-off between material properties and thickness in response to predator presence, where one defense mechanism is preferred over another as we hypothesized, but use both mechanisms depending on age as a method to balance the benefits over the costs of defense.

Alterations to shell thickness in response to predator cues are well documented among bivalves (Leonard et al., 1999; Freeman and Byers, 2006; Scherer et al., 2018) and other shelled organisms such as snails (Trussell, 1996; Brookes and Rochette, 2007); however, the use of both thickening and hardening mechanisms as a defense has not been documented before. Thicker shells are highly effective at resisting fracture at the macro-scale (Zuschin and Stanton, 2001; Avery and Etter, 2006) or increasing predator handling time, subsequently decreasing predation risk (Boulding, 1984; Lord and Whitlatch, 2012; Lombardi et al., 2013). In the present study, older juveniles increased shell thickness across shell layers, as we hypothesized, while also increasing the hardness of their prismatic shell layer in response to induction. Using both shell thickening and hardening mechanisms to defend against predation in this vulnerable life stage implies that the benefits of these combined defenses, i.e. enhanced resistance to predation, outweighs the costs of defense, energetic or otherwise (Harvell, 1990). The most plausible explanation for the results in the present study is that induced oyster spat grew harder shells by changing the organic material of their shell. Organic content of the shell directly affects its flexibility and fracture resistance (Esteban-Delgado et al., 2008), is known to alter calcitic crystal hardness (Kim et al., 2016), and has been linked to material hardness in other bivalves (Zuschin and Stanton, 2001). Other factors that could alter shell hardness, with varying costs, include the polymorph of CaCO_3_ used by the animal, organic inclusions or trace mineral (e.g. magnesium) content within calcite crystals (Kunitake et al., 2012; Rae Cho et al., 2016), or microstructural arrangement of the material (Beniash et al., 2010), though investigation of these features is outside the scope of this study. The use of both thickening and hardening mechanisms in response to induction is somewhat surprising given our understanding of the high energetic costs of producing organic material, and unlike Scherer et al. (2018), we did not find that 8-week-old oysters traded one mechanism for another. Given the efficacy of both increased shell thickness (Avery and Etter, 2006; Belgrad et al., 2021) and hardness (Zuschin and Stanton, 2001) at reducing predation risk via shell strengthening, it would appear that the benefits of defense significantly outweigh the costs of this investment and that compounding these defenses is highly advantageous to juvenile oysters.

Younger (4-week-old) oyster juveniles in this study did not experience any significant differences in shell thickness or micromechanical properties owing to induction; however, macro-scale shell strength assessments in oysters from the same experimental exposure showed that induced oysters were 41% stronger than non-induced oysters of the same age (Belgrad et al., 2021). This suggests that predator-cue induction must be altering macro-scale shell strength in 4-week-old oyster juveniles some other way. Trends in the data from the present study suggest the prismatic layer was marginally thicker and harder in induced oysters of the younger age group compared with non-induced oysters. We measured these aspects of shell thickening and hardness in two dimensions, but it is possible that small changes to thickness occur using a three-dimensional mechanism, given that forces from predators in nature propagate in three dimensions. However, any three-dimensional changes are undetectable in the present study. It is also possible that measured differences in shell micro-properties were not statistically significant owing to variability in the individual mechanisms younger oysters use to strengthen shells due to the duration of cue exposure. In the present study, 4- and 8-week-old juveniles were exposed to cues continuously post-settlement, meaning we cannot separate the effects of age from the duration of cue exposure. Younger oysters may require a longer cue-exposure time in order to fully respond to predator cues, especially given the energetic investment required for defense. Previous studies have shown that juvenile oysters have no response to predator cues when exposed at a younger age for 2 weeks compared with the same amount of exposure an older age (Eason et al., 2021), suggesting that the costs of defense investment are greater than the benefits at this young age. Given this is an age with significant developmental changes and that the costs of induction are energetically expensive, it is possible that a greater length of cue exposure is necessary to confirm the risk of predation and balance the costs with the benefits. This likely leads to high variation in oyster response, where some individuals respond to cues quicker than others.

The use of compounded mechanisms of defense in juvenile oysters via deposition of harder and thicker material may also be due to potential limitations in oyster shell size resulting from utilizing only a shell thickening response. Many shelled organisms experience limited tissue growth when paired with increased shell thickness, a concept known as the skeleton limitation hypothesis (Palmer, 1981). This hypothesis suggests that thickness and tissue/shell size cannot be simultaneously increased, and increasing shell thickness constrains the animal's ability to increase shell size. Size is highly important for prey selection by many predator types, including crabs (Eggleston, 1990) and oyster drills (Pusack et al., 2018), where these predators preferentially select smaller oysters (Toscano and Griffen, 2012). Therefore, it is highly beneficial for oysters to invest in increasing the total surface area of their shell when they are very small, seeking refuge from predation by altering size, but less beneficial when oysters are of a large enough size to reduce predation risk (i.e. older). Many studies show that induced oyster spat grow larger shells compared with non-induced oysters (Robinson et al., 2014; Scherer et al., 2016; Roney et al., 2023); however, this effect can vary. For example, Belgrad et al. (2021) found that 4-week-old spat-on-shell were 10% larger when exposed to predator cues, whereas 8-week-old oysters spat-on-shell, conversely, were 10% smaller when induced compared with controls of the same age. This suggests that 4-week-old oysters may prioritize growth in shell size as a defense over shell thickness, given the constraints of increasing both size and thickness simultaneously. These limitations may bias younger oysters toward defense mechanisms that do not involve increasing thickness. Older oysters may then increase shell thickness and hardness as defenses when size no longer changes their predation risk significantly. Eight-week-old oysters of both induced and non-induced groups in this study were, on average, 20 mm long, a size class similar to those less vulnerable to crushing predators (Eggleston, 1990). Given the risk-reducing size of 8-week-old oysters, these animals may use a combination of increases to shell thickness and hardness to improve overall shell strength, balancing the costs to result in a combined use of these defense mechanisms. This may also explain the age-dependent foliated layer hardness in juvenile oysters, where 4-week-old oysters were significantly harder than their 8-week-old counterparts. Higher costs of shell thickening due to the trade-off with tissue growth and size in younger oysters may result in a greater benefit for increasing foliated layer hardness to defend against predators. It is possible that as these juveniles age and grow, the negative consequences of thickness on tissue growth are decreased, allowing thickening to be incorporated as a defense. In summary, the benefits of increasing overall tissue mass and, subsequently, shell size can outweigh the benefits of increasing shell thickness in response to predation for younger and smaller oysters, resulting in a limited or highly variable defense response in 4-week-old oysters compared with those of a risk-reducing size, which use both mechanisms.

The size-dependent needs and constraints regarding tissue growth and shell formation indicate that vulnerable juvenile oysters must be highly selective in their mechanism of shell strengthening. Altering the hardness of shell material in response to predator cues, such as the prismatic layer in 8-week-old induced oysters, appears in tandem with fewer and smaller cracks, implying that this mechanism improves the shell material's resistance to breaking without sacrificing overall material integrity. In fact, altering the hardness of their prismatic layer in combination with increased shell thickness provides significant defense: 8-week-old induced oysters from the same broodstock were 63% stronger and had 300% higher survivorship compared with non-induced oysters of the same age in natural field conditions for 30 days (Belgrad et al., 2021). This result suggests that these compounded defense investments against predation are worthwhile even with higher energetic demands.

Mechanical properties of biological materials, including mollusk shell, are typically sensitive to hydration (Barthelat and Espinosa, 2007; Romana et al., 2013). To avoid potential variation in the extent of sample hydration from sample to sample and indent to indent during mechanical testing, samples were kept universally dry. In addition, it is possible that sample preservation procedures could affect mechanical properties of biological materials (Stefan et al., 2010). When possible, testing of mechanical properties immediately after collection is ideal. Logistically, however, this is not always feasible, particularly in studies that employ large sample sizes or multiple experimental treatments, or that leverage synergies in analytical techniques between geographically distant research laboratories. A technical limitation of the present study is that ethanol preservation was required to avoid tissue decay in the time between the conclusion of the experimental exposure and micromechanical analyses. To test the impact of ethanol preservation and sample hydration on juvenile oyster micromechanical properties, we conducted a series of complementary assessments of shells prepared directly from live animals and those from animals preserved in 70% ethanol (Supplementary Materials and Methods). Our supporting data suggest that micro-scale mechanical properties of juvenile oyster shells are not significantly affected by ethanol preservation (Fig. S1, Table S1). Sample hydration does have a slight, layer-specific effect on micromechanical properties, but importantly the interaction of preservation by hydration was not significant. This suggests that the effect of hydration is not influenced by ethanol preservation. Although these are limitations that could not be avoided in the study design, ethanol preservation is highly unlikely to have influenced our findings given the magnitude of the differences between predator-induced versus non-induced oyster spat, the consistency of preservation and testing methods across treatments, and the negligible effects of such preservation at the scales assessed in this study.

Many studies have documented the benefits of shell thickening mechanisms (Boulding, 1984; Zuschin and Stanton, 2001; Avery and Etter, 2006; Brookes and Rochette, 2007; Lombardi et al., 2013), and our findings, along with those of Belgrad et al. (2021), corroborate that shell thickness increases the ability of oysters to resist predation. Our finding that oyster juveniles use multiple defenses in order to strengthen their shell has greater implications for our understanding of the ontogenetic development of inducible defenses, specifically, the costs to fitness that may accompany them and how prey species balance these costs. Though we often expect significant consequences to be associated with inducible defenses in prey (Harvell, 1990), our findings suggest that organisms may experience these costs across many fine scales and balance them through different life stages while maintaining the appropriate defenses. This is indicated by the younger set of oysters in this study experiencing limited measurable alterations to the hardness or thickness of their shell material, with older juveniles later increasing overall shell thickness in combination with increased hardness. In general, the magnitude of defense costs varies and can influence the mechanism used by prey to decrease their predation risk, depending on the needs of their life stage. Other calcifying organisms likely face similar cost-balancing dilemmas, but current literature often focuses on exploring inducible defenses in a single life stage as opposed to sampling multiple ages of prey (Smith and Jennings, 2000; Lombardi et al., 2013; Corbett and Trussell, 2024). Further work exploring the mechanisms of defense across multiple life stages of prey organisms may reveal more information on the benefits of various mechanisms and the way individuals may balance the associated costs.

## Supplementary Material

10.1242/jexbio.250143_sup1 Supplementary information
