# Supplementary material for: Eastern oysters alter inducible defense mechanism of shell strengthening with age
Source: J Exp Biol. 2025 Jul 7;228(13):jeb250143. doi: 10.1242/jeb.250143 (PMC12276809; doi:10.1242/jeb.250143)
Supplement: Supplementary information [file jexbio-228-250143-s1.pdf]

Supplementary Materials and Methods

Assessment of ethanol preservation and hydration on oyster shell micromechanical properties

The oysters used in this study were stored in 70% ethanol and micromechanical testi g was conducted on embedded and polished shells when dry, following the methods of (Beniash et al. 2010) and Dickinson et al. (2012). Preserva on of oysters was necessary for our experimental approach, which employed a large number of replicate juveniles; testi g immediately a er the inducti n exposure was not logisti ally possible. Testi g all samples (induced and non-induced, 4-weeks and 8-weeks) uniformly in the dry condi on reduces potenti l for uctuatio in the level of hydratio from sample to sample and indent to indent.

To assess the e ect of ethanol preservation and sample hydra on on micromechanical proper es, we conducted a supplemental set of mechanical assessments with oysters that had either been prepared directly from live juvenile oysters or from animals that had been preserved in 70% ethanol for 8 weeks. Single seed eastern oysters were grown at the Auburn University Shellfish Laboratory on Dauphin Island, Alabama for four weeks without predator cues. Oysters were then raised in cages ‘o -bo om’ on French Hermit Oyster Farm in Biloxi Bay, Mississippi for ten weeks. At 14-weeks post-metamorphosis (from larvae to spat), oysters were split into two groups: ve individuals were placed into 70% ethanol and another ve were kept alive unti dissection. Live oysters were placed in a ~1,900L tank with recirculati g seawater (22 - 25°C, 20 ppt) for approximately 7 weeks; oysters were not fed during this ti e to curtail further growth, and 20-50% water changes were conducted weekly. Live oysters were then held moist at 4°C for approximately 1 week before dissecti n.

A er eight weeks, shell valves for ethanol-preserved and live oysters were separated, tiss e was removed, and le valves were trimmed (using a water-cooled diamond bandsaw; Gryphon C-40) to t into embedding cups. Sample embedding, polishing, and mechanical testi g followed the methods described in the main text. All samples were rst tested dry, within the prismati and foliated layers. Testi g methods followed those in the main text, with the excep on of a 10 g load used for the foliated layer. The same samples were then immersed in tap water for 48 hours. We ini ally rehydrated samples in ar cial seawater, but this procedure resulted in a precipitate that obscured imaging of indents made during mechanical tests. A er the samples had soaked for 48 hours, they were removed one at a ti e, and a series of indents were made as described in the main text. Indentations ere made as quickly as possible once the sample was removed from water (typically within 10 min) to prevent dehydration. Microhardness and crack propaga on data were assessed using a mixed-model ANOVA, with preserva on as the between-subject variable and hydration as the within-subject variable.

Ethanol preservation d d not a ect microhardness or crack propaga on in either the prismatic r foliated layers (Fig. S1; Table S1). Hydration resulted in a modest reduc on in microhardness (~19% lower in the prismatic layer when tested wet; 9% lower in the foliated layer). The length of cracks produced during

Journal of Experimental Biology • Supplementary information

mechanical testi g was higher when tested wet in the foliated layer, but did not di er between wet and dry samples within the prismatic layer. Importantly, the interac on of preserva on and hydration was not signifi ant for microhardness or crack propagation, in either the prismatic or foliated layers (Table S2). Altogether, these data suggest that use of ethanol-preserved shells is unlikely to a ect trends in micromechanical properti s observed and described in the main text.

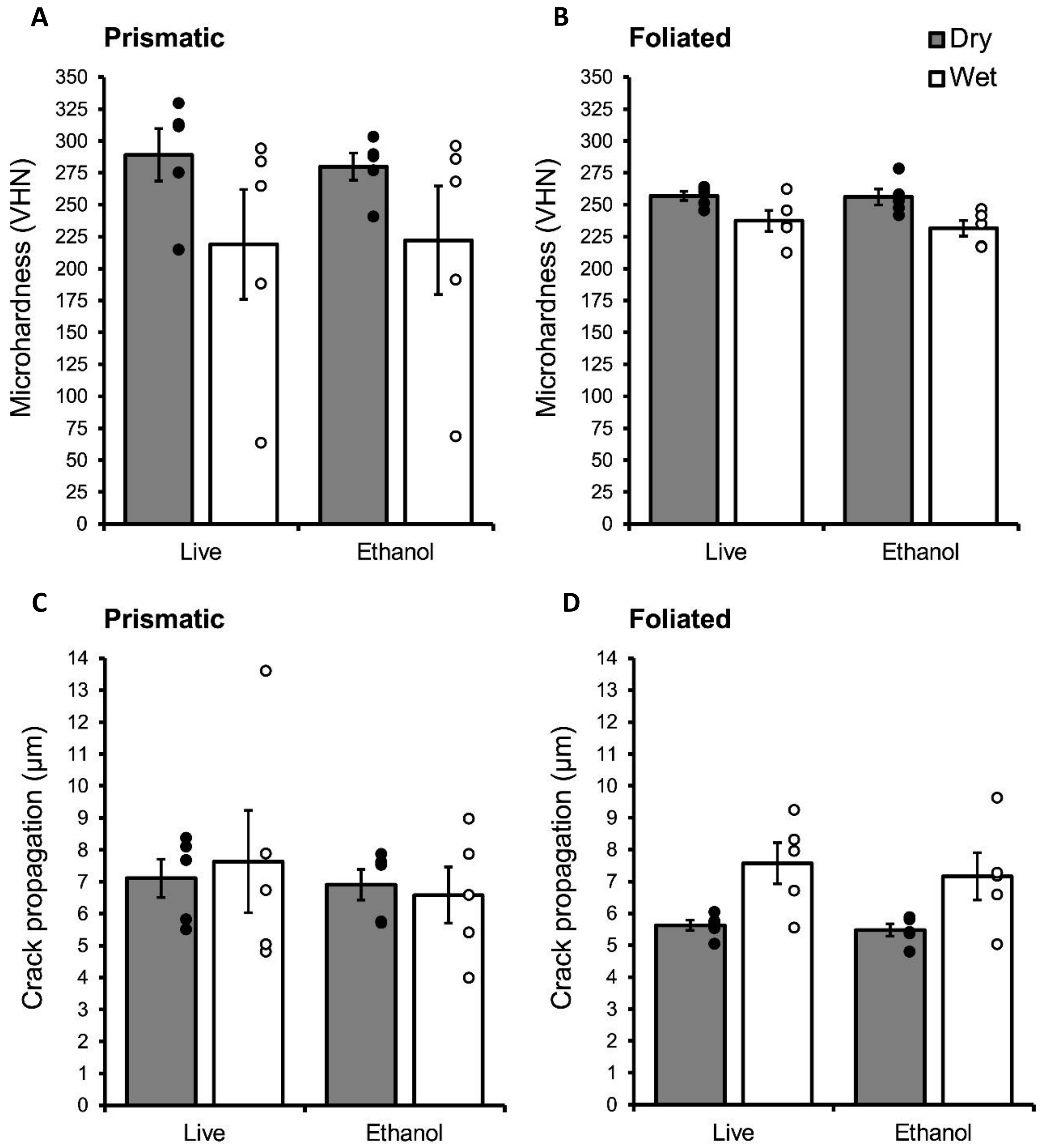

Fig. S1. Assessment of micromechanical properties in shells prepared directly from live juvenile eastern oysters (14 weeks post-metamorphosis) as compared to shells from animals that had been preserved in 70% ethanol (means ± SE; raw data are shown as points on each bar). Samples were tested in both the prismatic and foliated layers and all samples were tested dry and when wet. Preservation in ethanol did not affect microhardness or crack length in juvenile oyster shells for either the foliated or prismatic layer and the interaction of preservation and hydration was not significant (mixed-model ANOVA: $p >> 0.05$). $n = 5$ shells per treatment.

Journal of Experimental Biology • Supplementary information

Table S1. Mixed-model ANOVA table, assessing the effect of hydration and preservation on oyster shell mechanical properties.

	d.f.	*F*	*p*
**Prismatic layer**			
**Microhardness**			
Hydra on	1, 8	10.434	0.012
Preserva on	1, 8	0.005	0.944
Hydra on x Preserva on	1, 8	0.098	0.762
**Crack propagation**			
Hydra on	1, 8	0.012	0.917
Preserva on	1, 8	0.350	0.571
Hydra on x Preserva on	1, 8	0.213	0.657
**Foliated layer**			
**Microhardness**			
Hydra on	1, 8	22.434	0.001
Preserva on	1, 8	0.190	0.677
Hydra on x Preserva on	1, 8	0.274	0.615
**Crack propagation**			
Hydra on	1, 8	15.687	0.004
Preserva on	1, 8	0.261	0.624
Hydra on x Preserva on	1, 8	0.082	0.782

Table S2. The number of microhardness test replicates (VHN, crack length, and number of cracks) per shell for four-week-old and eight-week-old oysters of both induction states. Replicates were removed from all three analyses due to inappropriate shape or position on the shell layer during the testing process (ASTM Designation C1327–15R19, 2019) or if they were identified as outliers, defined as more than 1.5x outside the interquartile range.

	**Four Week**		**Eight Week**	
	Prismatic	*Foliated*	*Prismatic*	*Foliated*
**Induced**	8	10	10	10
	8	10	10	10
	8	10	10	10
	10	10	10	10
	9	10	10	10
	7	10	10	10
	10	10	9	10
	10	10	10	10
	8	9	9	10
	8	10	10	9
**Control**	7	10	10	10
	9	10	10	10
	8	10	10	10
	9	10	10	10
	9	10	9	10
	9	10	9	10
	8	10	9	10
	10	10	10	10
	7	10	8	10
	10	10	9	10

Journal of Experimental Biology • Supplementary information

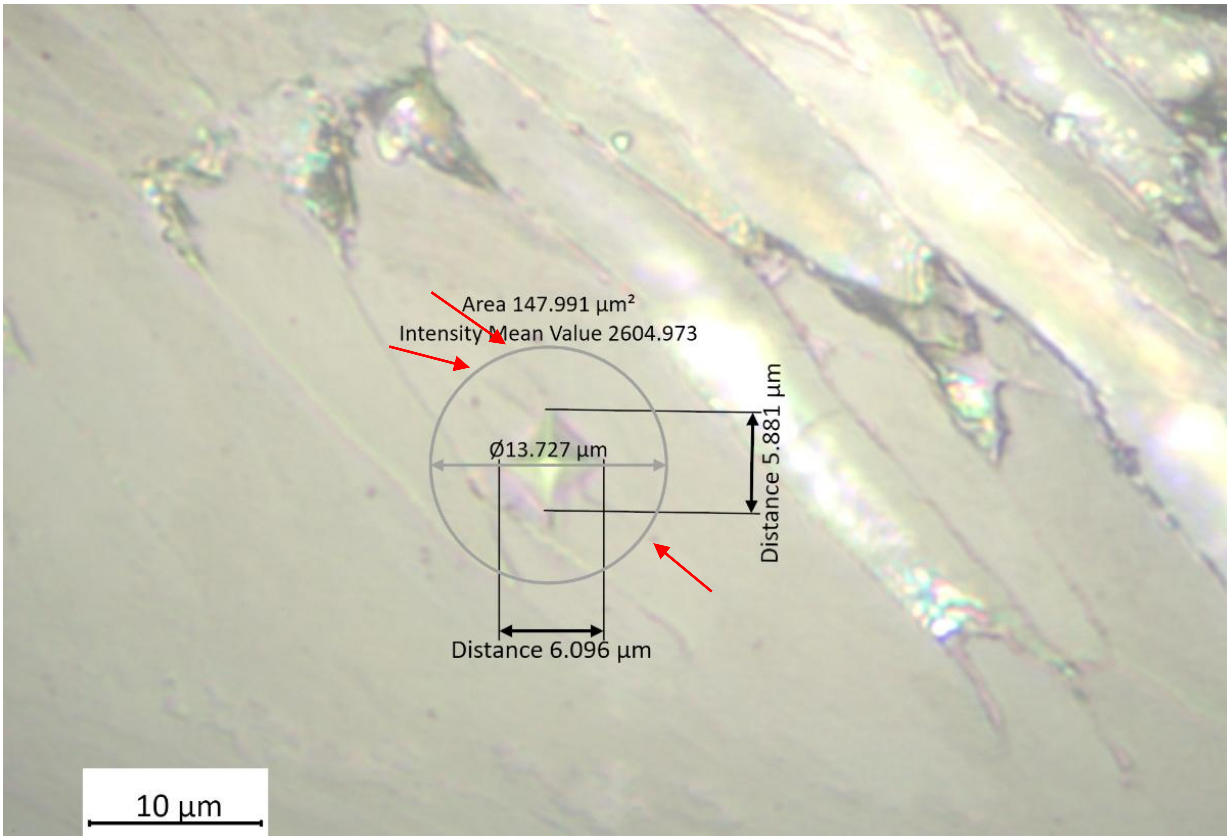

Fig. S2. Image of microhardness indent on the prismatic layer of an eight-week-old, predator induced oyster shell analyzed using Zeiss Zen 3.6 Blue software. The length (µm) of each indent diagonal (black lines) was measured and averaged together for the determination of Vickers microhardness number. The length (µm) of the longest crack originating from the microhardness (gray circle) indented was also measured. Cracks resulting from microhardness indent are indicated with red arrows.

Table S3. Mean ± SE values of each measurement on induced and non-induced, four week and eight week old eastern oysters in the study (n = 10 per age and induction state). Data is displayed in boxplots within the manuscript's main text.

	Four Week		Eight Week	
**Thickness**	Non-Induced	Induced	Non-Induced	Induced
Overall	114.7 ± 3.7	115.8 ± 2.2	296.5 ± 5.2	416.1 ± 6.4
Foliated	104.4 ± 4.7	95.3 ± 2.2	259.4 ± 4.9	361.7 ± 5.9
Prismatic	17.3 ± 0.6	18.7 ± 0.4	38.8 ± 1	54 ± 1.3
**Hardness**				
Foliated	267.4 ± 2.5	271 ± 2.6	253.2 ± 2.3	253.6 ± 2.6
Prismatic	218 ± 5.7	237.3 ± 6.2	234.5 ± 6.2	263.2 ± 4.6
**Number of Cracks**				
Foliated	1.8 ± 0.1	1.8 ± 0.1	1.9 ± 0.1	1.7 ± 0.2
Prismatic	3.5 ± 0.1	3.1 ± 0.1	3.4 ± 0.1	2.8 ± 0.1
**Crack Length**				
Foliated	9.7 ± 0.3	9.6 ± 0.3	9.8 ± 0.3	9.4 ± 0.2
Prismatic	22 ± 0.8	22 ± 0.9	19.7 ± 0.7	17.2 ± 0.5
**Size**	10.6 ± 0.75	11.1 ± 0.89	21.2 ± 1.2	20.14 ± 0.91
